# Working from home and subsequent work outcomes: Pre-pandemic evidence

**DOI:** 10.1371/journal.pone.0283788

**Published:** 2023-04-04

**Authors:** Ying Chen, Dorota Weziak-Bialowolska, Matthew T. Lee, Piotr Bialowolski, Richard G. Cowden, Eileen McNeely, Tyler J. VanderWeele

**Affiliations:** 1 Harvard Institute for Quantitative Social Science, Human Flourishing Program, Cambridge, MA, United States of America; 2 Department of Epidemiology, Harvard T.H. Chan School of Public Health, Boston, MA, United States of America; 3 Department of Environmental Health, Sustainability and Health Initiative (SHINE), Harvard T.H. Chan School of Public Health, Boston, MA, United States of America; 4 Faculty of Philosophy, Centre for Evaluation and Analysis of Public Policies, Jagiellonian University, Cracow, Poland; 5 Department of Economics, Kozminski University, Warsaw, Poland; University of Crete, GREECE

## Abstract

Frequent working from home (WFH) may stay as a new work norm after the COVID-19 pandemic. Prior observational studies on WFH and work outcomes under non-pandemic circumstances are mostly cross-sectional and often studied employees who worked from home in limited capacity. To provide additional insights that might inform post-pandemic work policies, using longitudinal data collected before the COVID-19 pandemic (June 2018 to July 2019), this study aims to examine the associations between WFH and multiple subsequent work-related outcomes, as well as potential modifiers of these associations, in a sample of employees among whom frequent or even full-time WFH was common (*N* = 1,123, *Mean*_age_ = 43.37 years). In linear regression models, each subsequent work outcome (standardized score was used) was regressed on frequencies of WFH, adjusting for baseline values of the outcome variables and other covariates. The results suggested that WFH for 5 days/week versus never WFH was associated with subsequently less work distraction (ß = -0.24, 95% CI = -0.38, -0.11), greater perceived productivity/engagement (ß = 0.23, 95% CI = 0.11, 0.36), and greater job satisfaction (ß = 0.15, 95% CI = 0.02, 0.27), and was associated with subsequent work-family conflicts to a lesser extent (ß = -0.13, 95% CI = -0.26, 0.004). There was also evidence suggesting that long work hours, caregiving responsibilities, and a greater sense of meaningful work can all potentially attenuate the benefits of WFH. As we move towards the post-pandemic era, further research will be needed to understand the impacts of WFH and resources for supporting employees who work from home.

## Introduction

The COVID-19 pandemic has led to unprecedented challenges to people’s social and work lives. To comply with the social distancing measures, 71% of the US workforce whose job could be mostly done from home worked remotely all or most of the time during the pandemic, whereas only 20% of them did so prior to the pandemic [1]. This wide-spread transition to working from home (WFH) may become a lasting consequence of the pandemic [2]. Some companies, such as Meta Platforms, have announced their long-term flexible remote work policy [3], and 54% of U.S. workers who could work from home indicated that they would want to continue WFH after the pandemic [1]. As we move toward the post-pandemic recovery era, it is important to understand how WFH impacts work outcomes under non-pandemic circumstances.

WFH (also referred to as telework, telecommuting, or remote work) is largely defined as “a work arrangement in which the work is done from places other than a traditional office space using information and communication technologies” [4]. WFH has become increasingly common over the past decade, but it generally has not been adopted as widely as possible prior to the COVID-19 pandemic. For example, around 56% of the full-time employees in the U.S. are remote-capable employees whose work can be done remotely from home, but as of 2019 only 42% of the U.S. workforce had ever worked from home [2,5]. WFH may have a broad range of implications for employees’ work and life. According to the Conservation of Resource Theory [6] and the Job Demands-Resources Model [7,8], WFH provides employees with resources for reducing work life conflicts (e.g., flexibility, autonomy), which may lead to greater work life balance, job satisfaction, and possibly higher productivity [9,10]. WFH may also help eliminate commute times which have been associated with various adverse health and well-being outcomes including poor mental health, poor diet, back pain, and cardiovascular disease [11,12]. Importantly, some studies have shown that the adverse health effects of commuting may be greater for women than men [13]. Not all studies demonstrate a negative impact of commuting on health, however. Recent data during the pandemic suggest some potential benefits of commuting, or venturing out versus staying home, related to ritualistic downtime, chance social encounters, and greater sense of purpose [14]. On the other hand, WFH has led to some unintended consequences for employees such as increased sense of isolation, distraction by family obligations, work intensification, longer work hours, reduced supervisor support and mentoring, and concerns about opportunities for promotion, all adding complexity to the impacts of WFH [4,9,15].

During the COVID-19 pandemic, the potential benefits of working in the office have also had to be weighed against pandemic-specific needs, such as reducing the risk of disease or being at home to manage uncertain events (e.g., school cancelations). Therefore, it is challenging to study the effects of “voluntary” WFH in the context of the pandemic. An emerging literature has sought to understand the impacts of “enforced WHF” during the pandemic [16,17]. However, the context of “enforced” (such as during the pandemic) and “voluntary” (such as prior to the pandemic) WFH may differ in many ways, such as employees’ motivations behind the choice of work arrangement, level of preparation for WFH, family arrangements, and sources of social interaction outside work [18]. It is, therefore, reasonable to expect that WFH may shape work outcomes differently during the COVID-19 pandemic than under normal circumstances.

Empirical studies on WFH prior to the pandemic are mostly cross-sectional observational studies, with the strongest evidence coming from only a handful of experimental or longitudinal observational studies. For instance, an experiment conducted in a sample of 249 employees at a call center at Ctrip (a 16,000-employee Chinese travel agency) in which participants were randomized to WFH or working in an office suggested that WFH was related to a 13% increase in productivity, substantially greater work satisfaction, and a reduced turnover rate by 50% over the 9-month study period [19]. Likewise, a natural experiment study among workers of U.S. Patent and Trademark Office [20] and three large-scale longitudinal panel studies in Europe [21–23] also found that WFH was related to higher productivity, greater job satisfaction, and reduced turnover intention. Further, a number of review studies and meta-analyses (though primarily based on cross-sectional evidence) also suggested that WFH generally has a positive impact on work outcomes, job attitudes, and work-family balance [16,24,25]. For instance, a meta-analysis by Gajendran et al. [24] based on 46 (mostly cross-sectional) studies involving 12,883 employees indicated that WFH was related to greater employee job satisfaction (r = 0.09), better supervisor-rated work performance (r = 0.18), and lower work-family conflicts (r = -.16), though the effect sizes were relatively small. In addition, there was evidence suggesting that the frequency of WFH matters. For instance, some studies have found that the association between WFH and job satisfaction follows an inverted U-shape, with the inflection point occurring when WFH exceeds 2 or 3 days per week [24,26]. Based on these findings, it was hypothesized that when telecommuting is extensive, there might be an increased likelihood of social isolation and sense of frustration that offset the benefits of increased autonomy [24,26].

While prior studies have substantially advanced our understanding of WFH, several knowledge gaps remain. For instance, the COVID-19 pandemic has shaped WFH practices in multiple aspects. Thus, WFH may follow new trends and adopt different patterns after the pandemic [2,27]. It is expected that as more companies allow WFH, workers may increasingly be WFH on a regular basis [1,28]. Prior work on pre-pandemic WFH has, however, often focused on workers who worked from home in limited capacity. It is, therefore, helpful to understand WFH in companies with a tradition of flexible work arrangements and among workers who have worked from home on a more continual basis. Moreover, heterogeneity in the association of WFH with work outcomes remains understudied. Such information would be helpful for identifying resources, informing training programs, and refining policies for supporting employees who are WFH. In addition, prior observational studies on “voluntary” WFH and work outcomes have mostly relied on cross-sectional data, and therefore are unable to address concerns about reverse causality. Although it may be unlikely that work outcomes would affect workers’ preferences to WFH, it is possible that managers may be more willing to provide WFH opportunities to high-preforming employees [24]. Longitudinal evidence would, therefore, be needed to help establish temporality.

To address some of these issues, the present study uses pre-pandemic longitudinal data from a sample of employees with a high rate of frequent “voluntary” WFH to examine the associations between a full spectrum of WFH frequencies and multiple work-related outcomes including work distraction, perceived productivity/work engagement, work-family conflict, and job satisfaction. These outcomes are commonly-used markers of employee work performance, work-family balance, and job attitudes, and together they represent the major aspects of employees’ work and life that may be impacted by WFH [16,25]. Based on prior evidence suggesting an inverted U-shaped association between WFH and some work outcomes [24,26], we hypothesized that moderate frequencies of WFH would be associated with better work performance and greater work-related wellbeing, as compared to lower or higher frequencies of WFH. Some prior evidence suggests that certain worker characteristics (e.g., family caregiving responsibilities, health status, purpose in life) and workplace resource factors (e.g., workload, coworker support, sense of meaningful work) may affect employees’ work outcomes [24,29–32], but their roles in the context of WFH remain unclear. To understand potential heterogeneity in the impacts of WFH on work outcomes, as a secondary analysis this study also explored a number of worker characteristics and workplace resources as potential modifiers of the associations.

## Methods

### Study participants

This study used data from a sample of employees at a large U.S. national self-insured service organization. At study baseline (June 2018), an invitation to participate was sent to a randomly selected sample of 15,000 employees in this organization through the work email system. A total of 2,364 employees returned the completed questionnaire (as an incentive for participation, 52 of them were randomly selected to win a cash prize ranging from $100 to $1000). In July 2019, participants who completed the initial survey and remained employed in the organization were asked to complete a follow-up survey, and 1,411 of them responded. The analytic sample for the present study was drawn from those who participated at both waves of data collection (N = 1,411). Those with missing data on WFH (n = 17), any of the work outcome variables (n = 177) or any covariates (n = 94) were excluded, yielding an analytic sample of 1,123 participants. As compared to those who were excluded, participants included in the sample were younger, worked from home less frequently, had shorter work hours, had greater self-rated physical health, reported lower coworker support, and perceived lower productivity/work engagement at baseline (S1 Table). However, participants in this sample were comparable to the total employee population in this organization on major sociodemographic characteristics such as gender, race/ethnicity, and education level [33]. This study was approved by the Institutional Review Board at Harvard T. H. Chan School of Public Health. Written informed consent was obtained from all participants.

### Assessment of the independent variable

#### Frequency of working from home (WFH)

Participants reported their frequency of WFH in response to the question at baseline: “How many days per week do you regularly work from home?”. Response categories ranged from 0 (0 days/week) to 5 (5 days/week). Because a majority of the participants in this sample reported either full-time WFH (5 days/week) or not WFH at all (0 day/week), to reduce data sparsity the responses were collapsed into three categories including 0 day/week, 1–4 days/week, and 5 days/week. In a sensitivity analysis, we also considered a more nuanced categorization scheme of 0 day/week, 1 day/week, 2 days/week, 3 to 4 days/week, and 5 days/week.

### Assessment of the dependent variables

#### Work distraction

Participants reported their assessment of distraction at work in response to the question: “Thinking about your last week of work, what percent of the time did you feel distracted or not as productive as you would like?” [34]. The response categories included 0% of time, 5–10% of time, 10–25% of time, 25–50% of time, and 50–100% of time. Following the approach in prior work [34], we took the mid-point of the categories as the response value (i.e., 0%, 7.5%, 17.5%, 37.5%, 75%), and considered them as a continuous score.

#### Perceived productivity/work engagement

The participants reported the extent to which they agreed with the following statement on work productivity/engagement: “The employees are productive and engaged” [35]. Response options ranged from 0 (strongly disagree) to 10 (strongly agree). The response was considered as a continuous score.

#### Work-family conflict

Work-family conflict was measured with an item from the previously-validated Work-Family Conflict Scale [36,37]: “Demands of my job interfere with my home life”. Response options ranged from 0 (strongly disagree) to 10 (strongly agree). The response was considered as a continuous score.

#### Job satisfaction

Job satisfaction was measured with a previously validated item: “How satisfied are you with your job?” [38–40]. The response ranged from 0 (strongly disagree) to 10 (strongly agree), and was used as a continuous score.

### Assessment of candidate modifiers and other covariates

Prior evidence has suggested that employee characteristics (e.g., sociodemographic factors, household responsibilities, psychosocial wellbeing, health status) and workplace resources (e.g., manageable workload, meaningful work, work/life integration, coworker support) may shape employees’ work outcomes in the traditional circumstances of working in an office [29–32]. However, their roles in the context of WFH are less clear. To understand potential heterogeneity in the impacts of WFH, we explored a number of such factors (as described in detail below) assessed at baseline as potential modifiers of the associations between WFH and subsequent work outcomes.

### Worker characteristics

Family responsibilities may interfere with one’s work, and women generally take more household responsibilities than men [41]. Therefore, we examined gender (male, female) and several indicators of family caring responsibilities, including caregiving to children (the number of children under the age of 18 that the participant took care at home, used as a continuous score), to elder persons (the number of elder parents or relatives that the participant took care at home, used as a continuous score), and to pets (pet ownership, yes or no), as modifiers. In addition, because psychological states may drive one’s work outcomes [29], we also examined sense of purpose in life (assessed with “My life has a clear sense of purpose” [42] on a scale from 0 [strongly disagree] to 10 [strongly agree]) as a modifier.

### Workplace resources

Prior studies have identified a number of workplace resources as drivers of employee performance and wellbeing, such as manageable workload, meaningful work, work recognition, and coworker support [31,32,35,43–45]. Therefore, this study explored these factors as candidate modifiers of the outcomes. First, work hours were examined as an indicator of workload with the question: “During a typical work week, about how many hours per day do you usually work?”. Response categories included “<8 hours”, “8 hours”, “9–10 hours”, and “>10 hours”. Second, participants used an 11-point response scale (0 = strongly disagree; 10 = strongly agree) to rate the extent to which they agreed with statements about meaningful work (i.e., “I find my work meaningful”) [46], work recognition (i.e., “I feel recognized for my work”) [47], and coworker support (i.e., “I feel part of a team at work”) [48].

### Other covariates

In addition to the abovementioned candidate modifiers, a number of other covariates were considered. These included sociodemographic characteristics including participants’ age (≤30 years, 31–50 years, >50 years), race/ethnicity (non-Hispanic White, other races/ethnicities), marital status (married or in partnership, unmarried), educational attainment (high school diploma or equivalent, some college, college degree, graduate school), and house ownership (yes, no). Because mental and physical health status may affect individual’s work outcomes and work-related wellbeing [49–51], we also adjusted for participants’ depressive symptoms (on a scale from 0 [not at all depressed] to 10 [very depressed]) and self-rated physical health (on a scale from 0 [poor] to 10 [excellent]) assessed at baseline. To reduce potential reverse causation, baseline values of all outcome variables (i.e., work distraction, productivity/ engagement, work-family conflict, job satisfaction) were additionally controlled for.

### Statistical analyses

Statistical analyses were performed in SAS 9.4 (SAS Institute Inc). Tests of statistical significance were two-sided. Analysis of variance and Chi-square tests were performed to examine participants’ baseline characteristics across frequencies of WFH.

Linear regression models were used in the primary analyses to regress each of the 4 work outcome variables (one at a time) at follow-up on frequencies of WFH at baseline, adjusting for baseline values of all the dependent variables simultaneously in addition to other covariates. The normality assumption of the linear regression models was tested, and there was no evidence suggesting violation of the assumption. For easier interpretation, the dependent variables were standardized (mean = 0, standard deviation = 1), such that the effect estimates were reported in terms of standard deviations in the dependent variables. As a sensitivity analysis, the primary sets of models were reanalyzed with a more nuanced categorization of the frequencies of WFH.

The secondary analyses explored candidate modifiers of the associations between WFH and work outcomes. Specifically, the primary sets of regression models were reanalyzed with an interaction term added between each of the candidate modifiers (examined one at a time) and the independent variable of WFH for each dependent variable.

To account for multiple testing, Bonferroni correction was applied. However, the practices for multiple testing vary in the literature and it is an evolving area of research [52,53]. Therefore, we do not use Bonferroni correction as the primary lens for interpreting the results. However, to acknowledge the different practices and types of cutoffs that can be used for interpreting the results, we reported our results both with and without Bonferroni correction and with multiple p-value cutoffs marked.

## Results

### Participant characteristics

The participants had a mean age of 43.37 (SD = 10.48) years, and were predominantly female (84.15%), non-Hispanic White (74.62%), married or in partnership (70.61%), had a college degree or higher (69.19%), owned a house (71.95%), and had caring responsibilities (to children, older persons, or pets) at home. A majority of the participants worked exclusively from home for 5 days/week (42.56%), and the remaining worked from home for 1 to 4 days/week (26.80%) or did not work from home at all (30.63%) (S1 Table). At the follow-up wave, the participants generally reported low levels of work distraction (over 75% of the participants reported being distracted for less than 10% of their time at work) and work family conflicts (mean = 3.20 out of a range of 0 to 10, SD = 2.94), as well as moderate levels of perceived productivity/work engagement (mean = 7.49 out of a range of 0 to 10, SD = 1.96) and job satisfaction (mean = 7.25 out of a range of 0 to 10, SD = 2.10).

As compared to those who did not work from home, participants who worked from home at least 1 day/week were more likely to be older, female, non-Hispanic White, married or in partnership, highly educated, and house-owners. They also tended to report fewer depressive symptoms, better self-rated physical health, greater purpose in life, a greater sense of meaningful work, shorter work hours, and greater job satisfaction at baseline (Table 1).

**Table 1:** Participant baseline characteristics by frequencies of work from home (N = 1,123).

Participant characteristics at baseline	Work from Home			
	0 day/week (n = 344)	1–4 days/week (n = 301)	5 days/week (n = 478)	*P*-value
	Mean (SD) or %	Mean (SD) or %	Mean (SD) or %	
Age groups, %				<0.001
≤ 30 years	23.26	14.29	3.56	
31–50 years	53.49	59.14	61.51	
>50 years	23.26	26.58	34.94	
Female, %	79.94	80.07	89.75	<0.001
Non-Hispanic White, %	66.57	75.08	80.13	<0.001
Married or in partnership, %	59.59	74.42	76.15	<0.001
Educational attainment, %				<0.001
High school diploma or equivalent	7.85	6.31	8.79	
Some college	34.01	15.28	19.87	
College degree	44.48	53.49	47.49	
Graduate degree	13.66	24.92	23.85	
House owner, %	54.36	74.42	83.05	<0.001
Depressive symptoms (range: 0 to 10)	2.33 (2.61)	1.71 (2.07)	2.08 (2.45)	0.005
Self-rated health (range: 0 to 10)	5.69 (1.86)	6.01 (1.60)	5.96 (1.79)	0.04
Number of children to take care at home (range: 0 to 5)	0.87 (1.14)	0.91 (1.11)	0.84 (1.08)	0.69
Number of older persons to take care at home (range: 0 to 2)	0.33 (0.61)	0.40 (0.67)	0.36 (0.62)	0.31
Pet owner, %	65.12	65.12	73.01	0.02
Sense of purpose in life (range: 0 to 10)	7.55 (2.06)	7.98 (1.72)	7.90 (1.82)	0.01
Work hours, %				<0.001
<8 hours/day	1.74	1.66	0.63	
8 hours/day	60.76	44.52	49.37	
9–10 hours/day	22.97	42.86	38.91	
>10 hours/day	14.53	10.96	11.09	
Sense of meaningful work (range: 0 to 10)	7.28 (2.28)	7.58 (1.87)	7.72 (2.12)	0.01
Work recognition (range: 0 to 10)	6.76 (2.74)	7.17 (2.41)	7.01 (2.55)	0.12
Coworker support (range: 0 to 10)	7.47 (2.64)	7.69 (2.22)	7.68 (2.40)	0.39
Work distraction (range: 0 to 75%)	0.12 (0.14)	0.10 (0.11)	0.11 (0.12)	0.11
Productivity/work engagement (range: 0 to 10)	7.28 (1.92)	7.25 (1.79)	7.41 (1.88)	0.46
Work-family conflicts (range: 0 to 10)	2.72 (2.78)	3.73 (2.91)	2.79 (2.85)	<0.001
Job satisfaction (range: 0 to 10)	7.04 (2.26)	7.30 (1.77)	7.46 (2.03)	0.01

Note: Analysis of variance or Chi-square tests were used to examine the mean levels (standard deviation) of the characteristic or proportion of individuals within each work from home frequency categories with that characteristic.

### Work from home and subsequent work outcomes

There was a monotonic inverse association between WFH and work distraction. As compared to no WFH, WFH for 1–4 days/week (ß = -0.20, 95% confidence interval [CI] = -0.34, -0.06) and for 5 days/week (ß = -0.24, 95% CI = -0.38, -0.11) were both associated with subsequently less work distraction, adjusting for baseline levels of work distraction and other covariates. These associations remained *p* < .05 after Bonferroni correction (Table 2). Sensitivity analysis using a more nuanced categorization of WFH frequencies yielded similar results (S2 Table).

**Table 2:** The associations between work from home and subsequent work outcomes (N = 1,123).

Subsequent work outcomes	Work from home		
	0 day/week	1 to 4 days/week	5 days/week
	Reference	β (95% CI)	β (95% CI)
Work distraction	1.00	-0.20 (-0.34, -0.06)**	-0.24 (-0.38, -0.11)**
Productivity/work engagement	1.00	0.02 (-0.12, 0.15)	0.23 (0.11, 0.36)**
Work-family conflicts	1.00	0.09 (-0.05, 0.23)	-0.13 (-0.26, 0.004)
Job satisfaction	1.00	-0.005 (-0.14, 0.13)	0.15 (0.02, 0.27)*

Note: Linear regression models were used to examine the association between work from home at baseline and each of the subsequent work outcomes at follow-up. All models controlled for age categories, gender, race/ethnicity, marital status, educational attainment, house ownership, depressive symptoms, self-rated health, number of children at home, number of older persons to take care at home, pet ownership, sense of purpose in life, work hours, sense of meaningful work, work recognition, coworker support, and the baseline values of all dependent variables (i.e., baseline values of work distraction, productivity/work engagement, work-family conflicts, and job satisfaction). All of the dependent variables were standardized at mean = 0 and standard deviation = 1.

*p<0.05 before Bonferroni correction

**p<0.05 after Bonferroni correction (The *p* value cutoff for Bonferroni correction is p = 0.05/4 outcomes = 0.0125).

As compared to never WFH, WFH for 5 days/week was associated with subsequently greater perceived productivity/work engagement (ß = 0.23, 95% CI = 0.11, 0.36), and the association remained *p* < .05 after Bonferroni correction (Table 2). However, less frequent WFH for 1–4 days/week did not differ from never WFH in perceived productivity/engagement. Results were similar when a more nuanced categorization of WFH frequencies was used (S2 Table).

The participants who worked exclusively from home reported subsequently greater job satisfaction (ß = 0.15, 95% CI = 0.02, 0.27), though the associations did not remain *p* < .05 after Bonferroni correction (Table 2). There was no difference between job satisfaction of participants with 1–4 days/week WFH and 0 day/week WFH. The sensitivity analysis with a more nuanced measure of WFH frequencies again yielded similar results (S2 Table).

The association of WFH with subsequent work-family conflicts was slightly weaker compared to the other outcomes, and did not meet the conventional *p* < .05 threshold before Bonferroni correction (Table 2). Sensitivity analysis with a more nuanced measure of WFH frequencies yielded similar results (S2 Table).

### Modifiers for the associations between WFH and work outcomes

There was generally little evidence that the candidate modifiers altered the associations. There were, however, a few exceptions (Table 3). Specifically, the inverse association of WFH with less work distraction was weaker among participants with caring responsibilities at home (specifically, caregiving to older persons or pets, but not to children). For instance, the inverse association between WFH (5 days/week vs. 0 day/week) and work distraction was substantially weaker among those who did versus did not have caregiving responsibilities to older people at home. Likewise, the inverse association with work distraction was weaker among participants with long work hours. However, the interaction terms of WFH with both caregiving responsibilities and work hours only passed the conventional, but not the Bonferroni-corrected *p* < .05 thresholds. Interestingly, the association with greater perceived productivity/engagement was weaker among those with a higher vs. lower sense of meaning of work, though the interaction term again only passed the conventional but not the Bonferroni-corrected *p* < .05 thresholds. There was, however, no substantial evidence that any of the candidate modifiers modified the associations of WFH with subsequent work-family conflicts or job satisfaction (Table 3).

**Table 3:** Interaction terms between work from home and candidate modifiers for the associations between work from home and subsequent work outcomes (N = 1,123).

	Work from home	
	1 to 4 days vs. 0 day /week	5 days vs. 0 day /week
	β (95% CI)	β (95% CI)
*Work distraction as the dependent variable*		
**Candidate modifiers**		
Gender (female vs. male)	-0.05 (-0.39, 0.30)	0.13 (-0.22, 0.48)
Caregiving to children at home (yes vs. no)	0.04 (-0.24, 0.31)	0.03 (-0.22, 0.27)
Caregiving to older persons at home (yes vs. no)	0.40 (0.09, 0.70)*	0.28 (0.005, 0.56)*
Pet ownership (yes vs. no)	0.27 (-0.02, 0.55)	0.30 (0.03, 0.57)*
Purpose in life (continuous)	-0.02 (-0.15, 0.12)	0.04 (-0.07, 0.16)
Work hours (continuous)	0.21 (0.02, 0.40)*	0.21 (0.04, 0.38)*
Meaningful work (continuous)	0.02 (-0.12, 0.16)	0.09 (-0.03, 0.20)
Work recognition (continuous)	-0.05 (-0.19, 0.09)	-0.001 (-0.12, 0.12)
Coworker support (continuous)	-0.10 (-0.24, 0.04)	-0.03 (-0.15, 0.09)
*Productivity/work engagement as the dependent variable*		
**Candidate modifiers**		
Gender (female vs. male)	0.06 (-0.26, 0.39)	0.08 (-0.25, 0.41)
Caregiving to children at home (yes vs. no)	-0.05 (-0.31, 0.21)	0.02 (-0.21, 0.26)
Caregiving to older persons at home (yes vs. no)	0.06 (-0.23, 0.35)	0.004 (-0.26, 0.27)
Pet ownership (yes vs. no)	-0.08 (-0.35, 0.20)	-0.05 (-0.30, 0.20)
Purpose in life (continuous)	-0.03 (-0.16, 0.10)	-0.01 (-0.12, 0.10)
Work hours (continuous)	0.09 (-0.09, 0.27)	0.02 (-0.14, 0.18)
Meaningful work (continuous)	-0.18 (-0.32, -0.05)**	-0.15 (-0.26, -0.04)**
Work recognition (continuous)	-0.03 (-0.16, 0.10)	-0.004 (-0.12, 0.11)
Coworker support (continuous)	-0.10 (-0.23, 0.03)	-0.05 (-0.16, 0.06)
*Work family conflicts as the dependent variable*		
**Candidate modifiers**		
Gender (female vs. male)	-0.18 (-0.52, 0.16)	0.06 (-0.29, 0.41)
Caregiving to children at home (yes vs. no)	0.20 (-0.08, 0.47)	-0.07 (-0.32, 0.18)
Caregiving to older persons at home (yes vs. no)	0.03 (-0.27, 0.34)	0.17 (-0.11, 0.45)
Pet ownership (yes vs. no)	0.28 (-0.01, 0.57)	0.26 (-0.01, 0.52)
Purpose in life (continuous)	-0.07 (-0.21, 0.06)	-0.04 (-0.16, 0.08)
Work hours (continuous)	0.01 (-0.18, 0.20)	-0.05 (-0.22, 0.12)
Meaningful work (continuous)	-0.06 (-0.20, 0.09)	-0.02 (-0.14, 0.10)
Work recognition (continuous)	-0.02 (-0.15, 0.12)	0.05 (-0.07, 0.17)
Coworker support (continuous)	0.002 (-0.14, 0.14)	0.05 (-0.07, 0.16)
*Job satisfaction as the dependent variable*		
**Candidate modifiers**		
Gender (female vs. male)	0.04 (-0.28, 0.36)	0.01 (-0.32, 0.33)
Caregiving to children at home (yes vs. no)	0.06 (-0.20, 0.31)	0.11 (-0.12, 0.34)
Caregiving to older persons at home (yes vs. no)	0.01 (-0.28, 0.29)	-0.07 (-0.33, 0.19)
Pet ownership (yes vs. no)	-0.11 (-0.38, 0.16)	-0.11 (-0.35, 0.14)
Purpose in life (continuous)	-0.09 (-0.22, 0.04)	-0.09 (-0.20, 0.02)
Work hours (continuous)	-0.12 (-0.20, 0.15)	0.03 (-0.13, 0.18)
Meaningful work (continuous)	-0.09 (-0.22, 0.05)	-0.07 (-0.18, 0.04)
Work recognition (continuous)	0.08 (-0.05, 0.21)	-0.01 (-0.12, 0.10)
Coworker support (continuous)	-0.01 (-0.14, 0.12)	0.07 (-0.04, 0.18)

Note: Linear regression models were used to examine candidate modifiers for the associations between work from home and each subsequent work outcome at follow-up. All models included an interaction term between the candidate modifier (examined one at a time) and work from home, and controlled for age categories, gender, race/ethnicity, marital status, educational attainment, house ownership, depressive symptoms, self-rated health, number of children at home, number of older persons to take care at home, pet ownership, sense of purpose in life, work hours, sense of meaningful work, work recognition, coworker support, and baseline values of all dependent variables (i.e., baseline values of work distraction, productivity/work engagement, work-family conflicts, and job satisfaction). All dependent variables and continuous independent variables were standardized (mean = 0 and standard deviation = 1).

*p<0.05 before Bonferroni correction

**p<0.01 before Bonferroni correction; There was no association that passed the *p* value threshold after Bonferroni correction in this table.

## Discussion

Over recent years, there have been calls for creating a caring climate at the workplace that makes employees feel being treated with care, trust, fairness and respect [35]. A regenerative workplace may help improve employee’s motivation at work and enhance their flourishing in life, which in turn may also boost organizational business outcomes [45,54]. Allowing employees to work from home may be one of the workplace resources that contribute to creating a caring organizational climate.

Congruent with the majority of prior studies on pre-pandemic WFH, the present study adds to the evidence that WFH is positively associated with multiple work-related outcomes [25,55,56], and extends the literature with longitudinal data from a unique sample with a high rate of frequent or even full-time WFH under non-pandemic circumstances. For instance, a prior meta-analysis (of mostly cross-sectional studies on pre-pandemic WFH) suggested that WFH was positively associated with objectively measured work performance (*r* = 0.18, 95% CI = 0.08, 0.26) and with job satisfaction (*r* = 0.09, 95% CI = 0.07, 0.11). Likewise, prior studies on “enforced WFH” during the pandemic indicated that switching to WFH was linked with increased or at least unchanged productivity [57,58], despite a decline in the average productivity per hour which was partly due to increased work hours among remote workers [59]. In comparison, empirical evidence on the impacts of WFH on work-family conflicts has been less clear. For instance, while some early meta-analyses [24,60] suggested a modest cross-sectional association between WFH and lower work interference with family, the present study was more consistent with recent work using large-scale panel data that suggested little medium-term effects of WFH on employee work-life balance [61]. Considering frequencies of WFH, somewhat contrary to our original hypothesis and to some prior literature which has suggested an inverted U-shaped association between frequencies of WFH and work outcomes [24,26], in our sample full-time remote work was associated with the best performance and highest job satisfaction. This may be attributable to the characteristics of this sample. Specifically, all our participants were employees of an organization with a long-standing flexible work tradition, where management, technical, and infrastructure support for WFH was available, and there might be less stigma associated with frequent WFH. Working exclusively from home might also help establish a consistent routine that reduces the need to organize one’s schedule and helps enhance one’s sense of coherence and meaning [62]. In comparison, prior studies seldom included participants who regularly worked from home for more than 3 days/week. Therefore, this sample provided an unique opportunity to understand a fuller spectrum of the frequencies of WHF. Taken together, the findings of this study provide some evidence supporting the Conservation of Resource Theory [6], the Job Demands-Resources Model [7,8], and the Caring Climate Workplace Framework [35], and suggest that work life integration should be treated as a workplace resource that could potentially contribute to improved work performance and worker wellbeing.

Heterogeneity in the associations between WFH and employee work outcomes remained understudied in the literature. The exploratory analysis in this study suggested that some worker characteristics and workplace resources might modify the WFH—work performance association. For instance, it was previously hypothesized that WFH carried the risk of blurring the boundaries between work and family, which might compromise employee productivity [25]. Consistent with this hypothesis, in this sample the inverse association between WFH and work distraction was weaker among participants with caring responsibilities at home (particularly to older persons and to pets). There was, however, little evidence that having children was a strong modifier, which may be attributed to the availability of childcare and education services under normal circumstances. Evidence on WFH during the pandemic when childcare and education services were interrupted indeed suggested stronger evidence that having young children at home can negatively impact WFH productivity [63]. Although women generally have greater household responsibilities than men [64,65], previous research indicated that women tend to have similar, if not higher, productivity in the office relative to men [66]. Considering WFH, while evidence pointed to greater vulnerabilities to disruption at work among women than men during COVID-19 pandemic lockdowns [66], this study’s findings are consistent with pre-pandemic evidence that has found little gender gaps in perceived productivity. This trend might be related to a greater likelihood of sacrifice of leisure time for work among women versus men and again to the availability of childcare services [67]. This study also suggested novel evidence that the inverse association between WFH and work distraction might be weaker among participants with long working hours. This might be related to the fatigue and burnout of maintaining boundaries between work roles and household responsibilities while WFH [68]. Interestingly, there was also some evidence that the positive association of WFH with perceived productivity/engagement was weaker among those with a stronger sense of meaningful work. One possible explanation for this finding is that WFH may affect the depth and/or breadth of knowledge an employee has about the inner workings of the organization (e.g., daily activities of its employees), which could be especially impactful among those who find their work to be more meaningful because they typically have a better understanding of the organization and how they fit within it [69]. Therefore, employees engaged in meaningful work but are WFH might not have sufficient opportunities to acquire the kind of detailed organizational knowledge they may need in order to rate the productivity/engagement of the workforce more positively. There was, however, little evidence that any of the candidate modifiers modified the association with work-family conflicts or job satisfaction in this sample. It is possible that WFH may improve job satisfaction more unanimously, whereas the association with work-family conflicts may vary by factors not examined in this study due to a lack of data (e.g., self-efficacy, characteristics of the workspace at home).

This study has certain limitations. First, the participants were primarily female and non-Hispanic White, and most were client service workers. The results, therefore, may not be applicable to other populations or to other job types. Second, the dependent variables and candidate modifiers in the study were mostly assessed with self-reported single-item questions. However, these items are all face valid and some of them were taken from previously validated scales. There was also prior evidence supporting the validity of single-item measures of worker wellbeing [38–40,70] and the validity of self-reported work performance [71]. In a prior meta-analysis (of mostly cross-sectional studies), WFH had an even stronger association with objectively-measured work performance outcomes compared to self-assessed performance [24]. Next, due to a lack of data, this study was not able to examine some potentially critical modifiers for the outcomes of WFH such as self-efficacy, or availability of a dedicated workspace at home [72,73]. Further, this study had only 1-year follow-up and was not able to examine the long-term impacts of WFH. These limitations are, however, balanced by important strengths of this study, such as its use of longitudinal data from a sample with a high rate of frequent and even full-time “voluntary WFH”, which provides a unique opportunity to understand the implications of frequent WFH under non-pandemic circumstances.

Taken together, this study adds to the evidence that WFH is positively associated with subsequent work-related outcomes under non-pandemic circumstances, and also suggests several workplace resources and worker characteristics as potential modifiers of the associations. The shift towards WFH induced by the COVID-19 pandemic is likely to stay as a new work norm long after the public health crisis wanes [58]. Although the pandemic has led to reduced stigma against WFH and a surge in investments enabling WFH, there are many challenges to and opportunities for improving the efficiency and sustainability of WFH [58,74]. Further research on the impacts of WFH on work and wellbeing under normal circumstances and potential resources for supporting WFH would be important for informing effective training programs and workplace innovations, which could help to cultivate and maintain a happy, healthy, and engaged workforce.

## Supporting information

S1 Table Comparison of baseline characteristics between the participants included in the analyses and those excluded from the analyses (N = 1,411). (DOCX)

S2 Table Sensitivity analysis on the associations between work from home and subsequent work outcomes (N = 1,123). (DOCX)
