# Supplementary material for: Working from home and subsequent work outcomes: Pre-pandemic evidence
Source: PLoS One. 2023 Apr 4;18(4):e0283788. doi: 10.1371/journal.pone.0283788 (PMC10072379; doi:10.1371/journal.pone.0283788)
Supplement: S1 Table — (DOCX) [file pone.0283788.s001.docx]

**S1 Table. Comparison of baseline characteristics between the participants included in the analyses and those excluded from the analyses (N=1,411).**

|  | Included  (n=1,123) | Excluded  (n=288) |
| --- | --- | --- |
|  | Mean (SD) or % | Mean (SD) or % |
| Work from home (days/week), % |  |  |
| 0 | 30.63 | 26.94 |
| 1 | 12.02 | 8.12 |
| 2 | 7.57 | 4.43 |
| 3 | 4.54 | 5.54 |
| 4 | 2.67 | 2.95 |
| 5 | 42.56 | 52.03 |
| Age groups, % |  |  |
| <=30 years | 12.47 | 6.60 |
| 31-50 years | 58.41 | 58.33 |
| >50 years | 29.12 | 35.07 |
| Female, % | 84.15 | 84.72 |
| Non-Hispanic White, % | 74.62 | 76.74 |
| Married or in partnership, % | 70.61 | 29.39 |
| Educational attainment, % |  |  |
| High school diploma or equivalent | 7.84 | 6.20 |
| Some college | 22.97 | 20.80 |
| College degree | 48.17 | 54.01 |
| Graduate degree | 21.02 | 18.98 |
| House owner, % | 71.95 | 76.75 |
| Depressive symptoms (range: 0 to 10) | 2.06 (2.41) | 2.17 (2.40) |
| Self-rated health (range: 0 to 10) | 5.89 (1.77) | 5.59 (1.81) |
| Number of children to take care at home (range: 0 to 5) | 0.87 (1.11) | 0.92 (1.12) |
| Number of older persons to take care at home (range: 0 to 2) | 0.36 (0.63) | 0.35 (0.62) |
| Pet owner, % | 70.07 | 68.48 |
| Sense of purpose in life (range: 0 to 10) | 7.81 (1.88) | 7.67 (2.10) |
| Work hours, % |  |  |
| <8 hours/day | 1.25 | 0.73 |
| 8 hours/day | 51.56 | 41.09 |
| 9-10 hours/day | 35.08 | 43.27 |
| >10 hours/day | 12.11 | 14.91 |
| Meaning of work (range: 0 to 10) | 7.55 (2.11) | 7.64 (1.99) |
| Workplace recognition (range: 0 to 10) | 6.98 (2.58) | 7.31 (2.63) |
| Coworker support (range: 0 to 10) | 7.62 (2.43) | 7.98 (2.30) |
| Work distraction (range: 0 to 75%) | 0.11 (0.12) | 0.11 (0.12) |
| Productivity/work engagement (range: 0 to 10) | 7.33 (1.87) | 7.59 (1.87) |
| Work family conflicts (range: 0 to 10) | 3.02 (2.88) | 3.43 (3.05) |
| Job satisfaction (range: 0 to 10) | 7.29 (2.04) | 7.44 (2.10) |

Note: The mean levels (standard deviation) of the characteristic or proportion of individuals within each inclusion status with that characteristic were examined.
