# Supplementary material for: Working from home and subsequent work outcomes: Pre-pandemic evidence
Source: PLoS One. 2023 Apr 4;18(4):e0283788. doi: 10.1371/journal.pone.0283788 (PMC10072379; doi:10.1371/journal.pone.0283788)
Supplement: S2 Table — (DOCX) [file pone.0283788.s002.docx]

**S2 Table. Sensitivity analysis on the associations between work from home and subsequent work outcomes with a nuanced measure of the frequencies of WFH (N=1,123)**

| **Work Outcomes** | **Work from Home** | | | |
| --- | --- | --- | --- | --- |
|  | 1 day vs 0 day/week | 2 days vs 0 day/week | 3 to 4 days vs 0 day/week | 5 days vs 0 day/week |
|  | β (95% CI) | β (95% CI) | β (95% CI) | β (95% CI) |
| Work distraction | -0.23 (-0.41, -0.05)* | -0.23 (-0.44, -0.01)* | -0.13 (-0.35, 0.10) | -0.24 (-0.37, -0.11)** |
| Productivity/work engagement | -0.03 (-0.20, 0.14) | 0.02 (-0.18, 0.23) | 0.08 (-0.13, 0.29) | 0.23 (0.11, 0.36)** |
| Work family conflicts | 0.11 (-0.07, 0.29) | 0.13 (-0.08, 0.35) | 0.003 (-0.22, 0.22) | -0.13 (-0.26, 0.002) |
| Job satisfaction | -0.06 (-0.22, 0.11) | 0.05 (-0.15, 0.25) | 0.03 (-0.18, 0.24) | 0.15 (0.02, 0.27)* |

Note: Linear regression models were used to examine the association between work from home at baseline and each of the subsequent work outcomes at follow-up. All models controlled for age categories, gender, race/ethnicity, marital status, educational attainment, house ownership, depressive symptoms, self-rated health, number of children at home, number of older persons to take care at home, pet ownership, sense of purpose in life, work hours, meaning of work, workplace recognition, coworker support, and the baseline values of all dependent variables (i.e., baseline values of work distraction, productivity/work engagement, work-family conflicts and job satisfaction). All dependent variables and continuous independent variables were standardized at mean=0 and standard deviation=1.

**p*<0.05 before Bonferroni correction; ***p*<0.05 after Bonferroni correction (The *p* value cutoff for Bonferroni correction is p=0.05/4 outcomes=0.0125).
